# *Verticillium*-like Anamorphic Fungi in Sordariomycetes from Southwestern China: Two New Taxa and a New Record

**DOI:** 10.3390/jof11080598

**Published:** 2025-08-18

**Authors:** Quan-Ying Dong, Shun-Yu Gao, Jin-Na Zhou, Cheng-Dong Xu, Zhen-Ji Wang, Nian-Kai Zeng

**Affiliations:** 1 College of Resources Environment and Chemistry, Chuxiong Normal University, Chuxiong 675000, China; nympheel@163.com (Q.-Y.D.); gsy88@cxtc.edu.cn (S.-Y.G.); zhoujinna@cxtc.edu.cn (J.-N.Z.); chtown@cxtc.edu.cn (C.-D.X.); 2 Ministry of Education Key Laboratory for Ecology of Tropical Islands, Key Laboratory of Tropical Animal and Plant Ecology of Hainan Province, College of Life Sciences, Hainan Normal University, Haikou 571158, China

**Keywords:** biocontrol, endophytes, new species, phylogeny, taxonomy

## Abstract

*Verticillium*-like fungi within the Sordariomycetes hold significant ecological and economic importance, especially in biocontrol. This study describes two novel species, *Leptobacillium gasaense* and *Ovicillium yunnanense*, and provides DNA sequence data and identification keys for the genera *Leptobacillium* and *Ovicillium*. The genus *Muscodor*, known for its considerable biotechnological value, comprises endophytes characterized by sterile mycelia that produce antibiotic volatile organic compounds (VOCs). Historically, the classification of *Muscodor* has relied on culture characteristics, VOC chemical profiles, and molecular phylogenetic analyses. However, culture characteristics and VOC profiles lack a definitive diagnostic value. Although asexual morphological traits are crucial for genus-level classification, no conidiogenous structures have been observed in *Muscodor*. Here, we report the asexual morphological characteristics of *Muscodor* and describe *M. coffeanus* as a new record in China, supported by both its asexual morphology and molecular phylogenetic evidence.

## 1. Introduction

*Verticillium*-like fungi are primarily characterized by their asexual stage, whose defining feature is the production of verticillate conidiophores. This group includes members of the orders Phyllachorales and Hypocreales [1]. With over 1000 recognized species across these orders, *Verticillium*-like fungi represent one of the largest groups of plant pathogenic fungi, exhibiting diverse parasitic interactions with hosts such as fungi, insects, nematodes, and rotifers. The asexual genera associated with *Verticillium*-like fungi include *Acremonium* Link, *Chlamydocillium* Zare & W. Gams, *Chlorocillium* Zare & W. Gams, *Cordyceps* Fr., *Engyodontium* de Hoog, *Hypomyces* Fr., *Lecanicillium* W. Gams & Zare, *Leptobacillium* Zare & W. Gams, *Marquandomyces* Samson et al., *Nectriopsis* Maire, *Ovicillium* Zare & W. Gams, *Pochonia* Bat. & O.M. Fonseca, *Simplicillium* W. Gams & Zare, *Sphaerostilbella* (Henn.) Sacc. & D. Sacc., and *Tolypocladium* W. Gams, among others [2,3,4,5,6,7,8,9].

Among the genera discussed here, *Muscodor* Worapong et al. represents the most prolonged and complicated nomenclatural history among the genera discussed here. *Induratia* Samuels et al., closely related to *Muscodor*, was initially described based on a single specimen, *Induratia apiospora* Samuels et al., collected from New Zealand. This species is characterized by uniperitheciate stromata bearing asci with an amyloid apical apparatus that produce apiosporous ascospores [10]. In subsequent mycelial cultures, a *Nodulisporium*-like conidial stage was observed, leading to the establishment of *Induratia* as a monotypic genus at that time. However, the genus received little attention in the following decades until Miller and Huhndorf [11] included a specimen labeled “*Induratia* sp. SMH 1255” from Puerto Rico in their phylogenetic study of Sordariales and other Sordariomycetes. Notably, neither the anamorph nor additional morphological data were provided for this specimen. Recently, Samarakoon et al. [7] obtained DNA sequences from this collection and supplemented them with a detailed sexual morph of the ascomata structures. These morphological features aligned with the original illustrations of *I. apiospora* by Samuels et al. [10], despite the loss of its holotype specimen. Nevertheless, the phylogenetic position of *I. apiospora* remains uncertain due to the lack of molecular data, although it has historically been classified within Xylariales. Molecular data from *Induratia* sp. SMH 1255 and two newly collected specimens from Thailand revealed a close relationship with the species of *Muscodor*, prompting the synonymization of *Induratia* and *Muscodor*. Under the principle of priority, the older name *Induratia* was retained, and a new family, Induratiaceae, was proposed, to include *Induratia*, *Emarcea* Duong, and the 25 species previously classified under *Muscodor* [12]. Subsequently, Cedeño-Sanchez et al. [13] re-evaluated the genus using the ex-holotype strain ATCC 60639 of *I. apiospora*. Through detailed morphological studies of the nodulisporium-like anamorph and multi-locus phylogenetic analyses, they reclassified *I. apiospora* within Barrmaeliaceae and synonymized Induratiaceae with Barrmaeliaceae. Additionally, they resurrected *Muscodor* within Xylariaceae and re-transferred the 25 species which were previously placed in *Induratia* back to *Muscodor*. *Muscodor* is one of the most biotechnologically significant genera in fungal taxonomy, comprising endophytic fungi that are endophytes on leaves, bark, and stems. These fungi are characterized by sterile mycelia that produce a diverse array of antibiotic volatile organic compounds (VOCs) [12,14,15,16]. Known as an endophytic fungal species with biocontrol potential, *M. albus* Worapong et al. was first described by Worapong et al. [17], as an endophyte isolated from small limbs of *Cinnamomum zeylanicum* Blume in Honduras; the culture showed strong antibiosis in dual culture with other microorganisms but did not sporulate [12].

The genus *Leptobacillium* was established with *L. leptobactrum* (W. Gams) Zare & W. Gams designated as the type species [6]. This taxon is morphologically characterized by predominantly solitary phialides with an extended morphology, irregularly branched conidiophores (rarely observed), and the production of distinctive narrow rod-shaped conidia. Members of this genus exhibit a broad ecological adaptability, having been isolated from diverse biotic and abiotic substrates including *Coffea arabica* Linn. branches, soil microbiota, fungal hosts, lepidopteran larvae, decaying wood, and environmental samples [6,18,19,20]. Currently, the genus comprises 12 recognized taxa: *L. cavernicola* Leplat, *L. chinense* (F. Liu & L. Cai) Okane et al., *L. coffeanum* (A.A.M. Gomes & O.L. Pereira) Okane et al., *L. filiforme* (R.M.F. Silva et al.) W.H. Chen et al., *L. latisporum* C. Srihom et al., *L. leptobactrum* (W. Gams) Zare & W. Gams with two varieties (*L. leptobactrum* var. *calidius* Zare & W. Gams and *L. leptobactrum* var. *leptobactrum*), *L. marksiae* Y.P. Tan, *L. muralicola* Jing Z. Sun, *L. symbioticum* Okane et al., and *L. xianyushanense* Ming J. Chen et al. [6,18,19,20,21,22,23,24].

Phylogenetically, *Leptobacillium* shares a sister relationship with *Simplicillium,* both of which are derived from reclassification of *Verticillium* Nees Section *Albo-erecta* [3,25]. Recent taxonomic revisions based on combined ITS and nrLSU sequence analyses prompted the transfer of two species (*S. chinense* F. Liu & L. Cai and *S. coffeanum A.A.M. Gomes* & *O.L. Pereira*) to *Leptobacillium* [19]. While the genera demonstrate overlapping phylogenetic positions in ITS-based trees, nrLSU sequence analyses provide a robust discrimination between them. Morphologically, *Simplicillium* species are distinguished by their prostrate growth habit and poorly differentiated conidiophores, typically producing solitary phialides directly from aerial hyphae with minimal structural complexity.

The genus *Ovicillium* currently includes seven recognized species: *O. asperulatum* (Giraldo et al.) L.W. Hou et al., *O. attenuatum* Zare & W. Gams (designated as the type species), *O. napiforme* Zare & W. Gams, *O. oosporum* Zare & W. Gams, *O. sinense* W.H. Chen et al., *O. subglobosum* Zare & W. Gams, and *O. variecolor* (Giraldo, Guarro, Gené & Cano) L.W. Hou et al. Members of this genus exhibit ecological versatility, having been isolated from diverse substrates across multiple biogeographical regions. Taxa within *Ovicillium* are principally characterized by their distinctive conidial morphology, typically producing subglobose to oval conidia as a key diagnostic feature [6,26,27]. However, despite these taxonomic advances, two critical knowledge gaps persist: (1) the absence of comprehensive phylogenetic frameworks integrating multi-locus data to resolve cryptic speciation and (2) the paucity of biodiversity records from East Asian subtropical forests harboring understudied fungal communities.

Fungi are keystone functional components in tropical rainforest ecosystems, playing critical roles in nutrient cycling and the ecological balance. However, a comprehensive understanding of their biodiversity patterns and biogeographical distribution remains fragmented across these megadiverse habitats. This investigation employed integrated morphological characterization and ITS and nrLSU sequence data of rainforest-derived specimens to systematically elucidate fungal diversity profiles in the understudied Southwest China ecoregion.

## 2. Materials and Methods

### 2.1. Specimen Collection and Preservation

Fungal specimens analyzed in this study were meticulously collected from their respective host organisms within community forests spanning diverse regions of China. Specimens were harvested using sterilized scoops under stringent aseptic conditions and promptly transferred into pre-sterilized collection bags to ensure the samples’ integrity and prevent contamination. Comprehensive metadata, encompassing precise geographical coordinates (latitude, longitude, and altitude) and detailed habitat characteristics, were meticulously documented for each collection site to establish the ecological context and facilitate future studies.

### 2.2. Fungal Isolation

Specimens were washed with tap water, surface-sterilized in 30% H_2_O_2_ (30–60 s), washed five times with sterile water, and dried on sterile filter paper. Insect tissue fragments were aseptically excised and transferred to potato dextrose agar for fungal isolation (PDA: 200 g/L potato, 20 g/L dextrose, 20 g/L agar), and then the plates were incubated at 25 °C, with purified strains maintained at 25 °C or on PDA slants at 4 °C [9].

Voucher specimens (accession series: CXTC 0001–0006) and associated axenic strains (accession series: CXCC 0001–0006) were deposited in the Chuxiong Teacher College Herbal Herbarium (CXTC) and Chuxiong Fungal Culture Collection (CXCC) at Chuxiong Normal University, China, for permanent archiving. This repository ensures accessibility for taxonomic validation, molecular studies, and future research.

### 2.3. Morphological Characterization

Ecological characteristics, including the host or substrate and fungi location, were documented. Cultures on slants were transferred to PDA plates and incubated for 14 days at 25 °C. Cultures were assessed for conidial arrangement, phialide morphology, and pigment production. For morphological evaluation, microscope slides were prepared by placing mycelia from the cultures on PDA blocks (5 mm diameter) overlaid with a coverslip and then cultivated in Petri dishes with a small amount of water. The sizes and shapes of the asexual morphological characteristics, including the conidiophores, phialides, and conidia, were determined using a light microscope (BX53, Olympus Corporation, Tokyo, Japan).

### 2.4. Extraction of DNA, Polymerase Chain Reaction (PCR), and Molecular Sequencing

Total genomic DNA was extracted from fungal mycelia grown on PDA plates using a Plant DNA Isolation Kit (FORE GENE, Chengdu, China) following the manufacturer’s protocol. The internal transcribed spacer (ITS) region was amplified using the primer pair ITS4 and ITS5 [28], while the nuclear ribosomal large subunit (nrLSU) was amplified using the primer pair LR5 and LR0R [29,30].

PCR reactions were performed in a final volume of 25 μL, containing 2.5 μL of 10× PCR Buffer (2 mmol/L Mg^2+^) (Transgen Biotech, Beijing, China), 0.25 μL of Taq DNA polymerase (Transgen Biotech, Beijing, China), 2 μL of dNTPs (2.5 mmol/L), 1 μL of DNA template (500 ng/μL), 1 μL of each forward and reverse primer (10 µmol/L), and 17.25 μL of sterile ddH_2_O. Amplification was carried out on a T100™ Thermal Cycler (BIO-RAD Laboratories, Hercules, CA, USA) using PCR programs as described by Dong et al. [9]. PCR products were purified using the Gel Extraction and PCR Purification Combo Kit (Beijing Genomics Institute, Shenzhen, China) and subsequently sequenced on an automated sequencer (BGI Co., Ltd., Shenzhen, China) with the same primers used for amplification.

### 2.5. Phylogenetic Analyses

Phylogenetic reconstruction was conducted using a two-locus framework integrating ITS and nrLSU sequences. Published sequences retrieved from GenBank were combined with novel sequences generated in this study, with full taxonomic details and corresponding accession numbers cataloged in Table 1. Sequence alignments were performed using MAFFT v.7.409 (http://mafft.cbrc.jp/alignment/server/, accessed on 1 February 2025) under default parameters, followed by manual refinement in BioEdit v7.2.6 to optimize positional homology. Following sequence alignment, the aligned sequences of two genes were concatenated. A partition homogeneity test (PHT; implemented in PAUP 4.0a166*) confirmed congruence between the two loci (*p* > 0.01), validating their combinability [31]. Optimal partitioning schemes and substitution models were determined via PartitionFinder2 v2.0.0 [32] under the Bayesian Information Criterion (BIC). Two partitions were defined: (1) ITS and (2) nrLSU. jModelTest2 v2.1.4 identified the GTR + G + I model as optimal for both partitions [33].

Maximum likelihood (ML) analysis was executed in IQ-TREE with partitioned model parameters and 1000 rapid bootstrap replicates to assess nodal support [34]. For Bayesian inference (BI), two independent runs of 5 million generations were performed in MrBayes v3.2.7 [33], sampling trees every 1000 generations following a 25% burn-in period. Convergence was monitored by calculating diagnostics every 10,000 generations and verifying effective sample sizes (ESS > 200) in Tracer v1.7.2. Final topologies from ML and BI analyses were visualized and annotated in FigTree v1.4.4.

**Table 1:** Specimen information and GenBank accession numbers for sequences used in this study.

Species	Strains	Host/Substrate	GenBank Accession Number		References
			ITS	nrLSU	
*Achaetomium macrosporum*	CBS 532.94	Mangrove mud	KX976574	KX976699	[35]
*Chaetomium elatum*	CBS 374.66	Decomposing leaf	KC109758	KC109758	[36]
*Leptobacillium cavernicola*	LRMH C212	Air	OM622523	OM628781	[20]
*L. cavernicola*	LRMH C299 ^T^	Surface sampling	OM622527	OM628786	[20]
*L. chinense*	NTUCC 20-073	–	MT974199	MT974414	[19]
*L. chinense*	LC 1345	Environmental microorganism	JQ410324	JQ410322	[18]
*L. chinense*	CGMCC 3.14970 ^T^	Environmental microorganism	–	NG069101	[18]
*L. coffeanum*	COAD 2057 ^T^	*Coffea arabica*	MF066034	MF066032	[21]
*L. coffeanum*	COAD 2061	*Coffea arabica*	MF066035	MF066033	[21]
*L. filiforme*	URM 7918	*Citrullus lanatus*	MH979338	MH979399	[37]
*L. latisporum*	TBRC 16288 ^T^	Soil	OP856540	OP856529	[23]
*L. leptobactrum var. calidius*	CBS 748.73 ^T^	Lepidopteran larva	EF641867	KU382227	[6]
*L. leptobactrum var. calidius*	CBS 251.81	Cyst of *Heterodera glycines*	KU382173	–	[6]
*L. leptobactrum var. calidius*	CBS 703.86	*Hemileia vastatrix* on *Coffea*	EF641866	KU382226	[6]
*L. leptobactrum var. calidius*	CBS 160.94	–	KU382172	–	[6]
*L. leptobactrum var. calidius*	CBS 109351	–	EF641863	–	[6]
*L. leptobactrum* var. *leptobactrum*	CBS 771.69	Soil under *Beta vulgaris*	–	KU382224	[6]
*L. leptobactrum* var. *leptobactrum*	CBS 774.69 ^T^	Decaying wood	KU382167	–	[6]
*L. leptobactrum* var. *leptobactrum*	CBS 775.69	*Lactarius rufus*	KU382170	–	[6]
*L. leptobactrum* var. *leptobactrum*	CBS 414.70	*Phlebia tremellosa*	KU382171	–	[6]
*L. leptobactrum* var. *leptobactrum*	CBS 305.93	Human nail	KU382169	–	[6]
*L. leptobactrum* var. *leptobactrum*	CBS 266.94	Human toenail	KU382168	–	[6]
*L. leptobactrum* var. *leptobactrum*	CBS 116723	Shrub sandy soil	EF641869	–	[6]
*L. leptobactrum* var. *leptobactrum*	IRAN 1230C	Unknown ascomycete	–	KU382225	[6]
***L. gasaense***	**CXCC 0003** ^T^	***Gibellula*** **sp.**	**PV037618**	**PV037622**	**This study**
***L. gasaense***	**CXCC 0004**	***Gibellula*** **sp.**	**PV037619**	**PV037623**	**This study**
*L. symbioticum*	Soy1-2 ^T^	Soybean leaf	LC485673	LC506046	[19]
*L. symbioticum*	OPTF00168	Soybean leaf	LC485675	LC506047	[19]
*L. symbioticum*	NBRC 104297	Soybean leaf	LC485674	AB378539	[19]
*L. xianyushanense*	RCEF6793	*Camellia oleifera* rhizosphere soil	OQ780699	OQ780702	[24]
*L. xianyushanense*	RCEF6795	*Camellia oleifera* rhizosphere soil	OQ780930	OQ780931	[24]
*Muscodor albus*	MONT 620 ^T^	*Cinnamomum zeylanicum*	AF324336		[38]
*M. albus*	9-6	–	HM034857	HM034865	[39]
*M. brasiliensis*	LGMF 1256 ^T^	*Schinus terebinthifolius* (Anacardiaceae)	KY924494	–	[14]
*M. camphorae*	NFCCI 3236 ^T^	*Cinnamomum camphora*	KC481681	–	[40]
*M. cinnamomi*	BCC 38842 ^T^	*Cinnamomum bejolghota* (Lauraceae)	GQ848369	–	[41]
*M. coffeanus*	COAD 1842 ^T^	*Coffea arabica*	KM514680	–	[42]
*M. coffeanus*	COAD 1900	*Coffea arabica*	KP862879	–	[42]
***M. coffeanus***	**CXCC 0001**	***Ophiocordyceps*** **sp.**	**PV037616**	**PV034610**	**This study**
***M. coffeanus***	**CXCC 0002**	***Ophiocordyceps*** **sp.**	**PV037617**	**PV034611**	**This study**
*M. crispans*	MONT 2347 ^T^	*Ananas ananassoides*	EU195297	–	[43]
*M. darjeelingensis*	NFCCI 3095 ^T^	*Cinnamomum camphora*	JQ409997	–	[44]
*M. equiseti*	JCM 18233 ^T^	*Equisetum debile* (Equsetaceae)	JX089322	–	[45]
*M. fengyangensis*	CGMCC 2862 ^T^	*Actinidia chinensis*, *Pseudotaxus chieni*	HM034856	HM034859	[39]
*M. fengyangensis*	CGMCC 2863	*Actinidia chinensis*, *Pseudotaxus chieni*	HM034855	HM034861	[39]
*M. ghoomensis*	NFCCI 3234 ^T^	*Cinnamomum camphora*	KF537625	–	[46]
*M. indicus*	NFCCI 3235 ^T^	*Cinnamomum camphora*	KF537626	–	[46]
*M. kashay*	NFCCI 2947 ^T^	*Aegle marmelos*	KC481680	–	[47]
*M. musae*	JCM 18230 ^T^	*Musa acuminata*	JX089323	–	[45]
*M. oryzae*	JCM 18231 ^T^	*Oryza rufipogon*	JX089321	–	[45]
*M. roseus*	MONT 2098 ^T^	*Grevillea pteridifolia* and *Erythophelum chlorostachys*	AH010859	–	[38]
*Muscodor* sp.	SMH 1255	Dead wood	MN250031	AY780069	[11,12]
*M. strobelii*	NFCCI 2907 ^T^	*Cinnamomum zeylanicum*	JQ409999	–	[48]
*M. suthepensis*	JCM 18232 ^T^	*Cinnamomum bejolghota*	JN558830	–	[45]
*M. suturae*	MSUB 2380 ^T^	*Prestonia trifida*	JF938595	–	[49]
*M. thailandicus*	MFLUCC 17-2669 ^T^	Dead wood	MK762707	MK762714	[12]
*M. thailandicus*	HKAS 102323	Dead wood	MK762708	MK762715	[12]
*M. tigerensis*	NFCCI 3172 ^T^	*Cinnamomum camphora*	JQ409998	–	[50]
*M. vitigenus*	MONT P-15 ^T^	*Paullinia paullinioides*	AY100022	–	[51]
*M. yucatanensis*	MEXU 25511 ^T^	*Bursera simaruba*	FJ917287	–	[52]
*M. yucatanensis*	CGMCC 3.18908 ^T^	*Oplismenus undulatifolius*	MG866046	MG866038	[53]
*M. ziziphi*	MFLUCC 17-2662 ^T^	*Ziziphus* sp.	MK762705	MK762712	[12]
*M. ziziphi*	HKAS 102300	*Ziziphus* sp.	MK762706	MK762713	[12]
*Ovicillium asperulatum*	CBS 426.95	Wood of Sorbus aria	KU382192	KU382233	[26]
*O. asperulatum*	CBS 130362 ^T^	Soil	OQ429756	OQ055655	[26]
*O. attenuatum*	CBS 399.86 ^T^	Dead mite on *Auricularia* sp.	OQ429757	OQ055656	[6]
*O. oosporum*	CBS 110151 ^T^	*Theobroma gileri*	OQ429758	OQ055657	[6]
*O. sinense*	SD09701 ^T^	Lepidoptera Pupa	PP836762	PP836764	[27]
*O. sinense*	SD09702	Lepidoptera Pupa	PP836763	PP836765	[27]
*O. subglobosum*	CBS 101963 ^T^	Soil	OQ429759	OQ055658	[6]
*O. variecolor*	CBS 130360 ^T^	Forest soil	OQ429760	OQ055659	[26]
***O. yunnanense***	**CXCC 0005** ^T^	**Dead insect on leaf**	**PV037620**	**PV037625**	**This study**
***O. yunnanense***	**CXCC 0006**	**Dead insect on leaf**	**PV037621**	**PV037626**	**This study**
*Simplicillium aogashimaense*	JCM 18167 ^T^	Soil	AB604002	NG068547	[54]
*S. calcicola*	CGMCC 3.17943 ^T^	Calcaire	KU746706	KU746752	[55,56]
*S. cicadellidae*	GY11011 ^T^	Cicadellidea	MN006243	–	[57]
*S. humicola*	CGMCC3.19573 ^T^	Soil	MK329136	MK329041	[58]
*S. lamellicola*	CBS 116.25 ^T^	*Agaricus bisporus*	AJ292393	AF339552	[55,59]
*S. lanosoniveum*	CBS 704.86	*Hemileia vastatrix*	AJ292396	AF339553	[55,59]
*S. lanosoniveum*	CBS 101267	*Hemileia vastatrix*	AJ292395	AF339554	[55,59]
*S. spumae*	JCM 39051 ^T^	From aquarium	LC496870	LC496884	[60]
*S. yunnanense*	YFCC 7133 ^T^	*Akanthomyces waltergamsii*	–	MN576784	[61]
*Sordaria fimicola*	CBS 508.50	–	AY681188	AF132330	[62]

Boldface: data generated in this study, ^T^ ex-type material, – means no data.

## 3. Results

### 3.1. Sequencing and Phylogenetic Analyses

**Figure 1:**
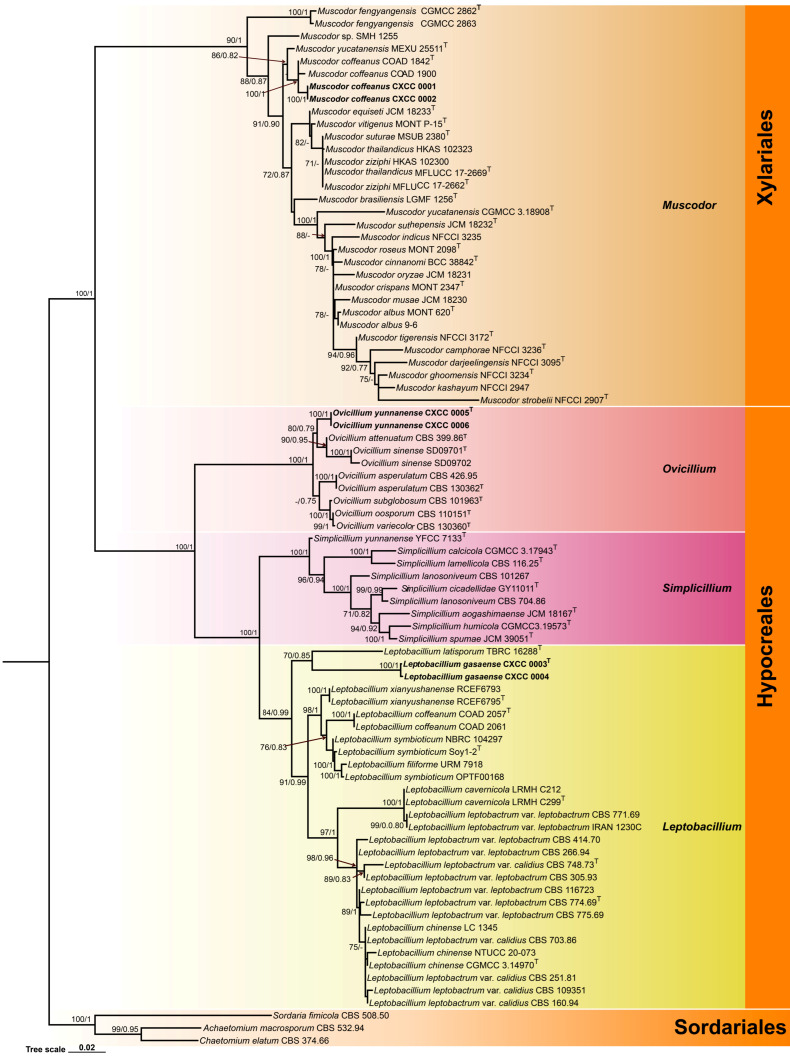
Molecular phylogenetic analyses using the ML and BI based on combined ITS and nrLSU sequence data. Three species in Sordariales (*A. macrosporum* CBS 532.94, *C. elatum* CBS 374.66, and *Sordaria fimicola* CBS 508.50) were used as outgroup taxa. Statistical support values (BS ≥ 70% and PP ≥ 0.70) are shown at the nodes for ML bootstrap support (BS) and BI posterior probabilities (PP). Isolates in bold type are those analyzed in this study.

The phylogenetic analysis of *Leptobacillium*, *Muscodor*, and *Ovicillium* species was conducted using a data matrix comprising sequences from 83 samples (Table 1). Three species of Sordariales (*Achaetomium macrosporum* CBS 532.94, *Chaetomium elatum* CBS 374.66, and *Sordaria fimicola* CBS 508.50) were utilized as outgroup taxa. The final dataset consisted of 1667 bp of sequence data, including gaps (ITS 791 bp and nrLSU 876 bp). Both BI and ML analyses generated trees with congruent topologies, resolving most *Muscodor*, *Ovicillium,* and *Leptobacillium* lineages into distinct terminal clades (Figure 1). The phylogenetic trees exhibited overall topologies consistent with previous studies [6,12,13,24,27,63]. The analyses also revealed that two newly discovered species, *L. gasaense* and *O. yunnanense*, were phylogenetically clustered with *L. latisporum, O. attenuatum*, and *O. sinense*. However, they were distinguished from the latter three by forming two separate branches in the *Leptobacillium* subclade and *Ovicillium* subclade (Figure 1).

### 3.2. Morphological Features

**Figure 2:**
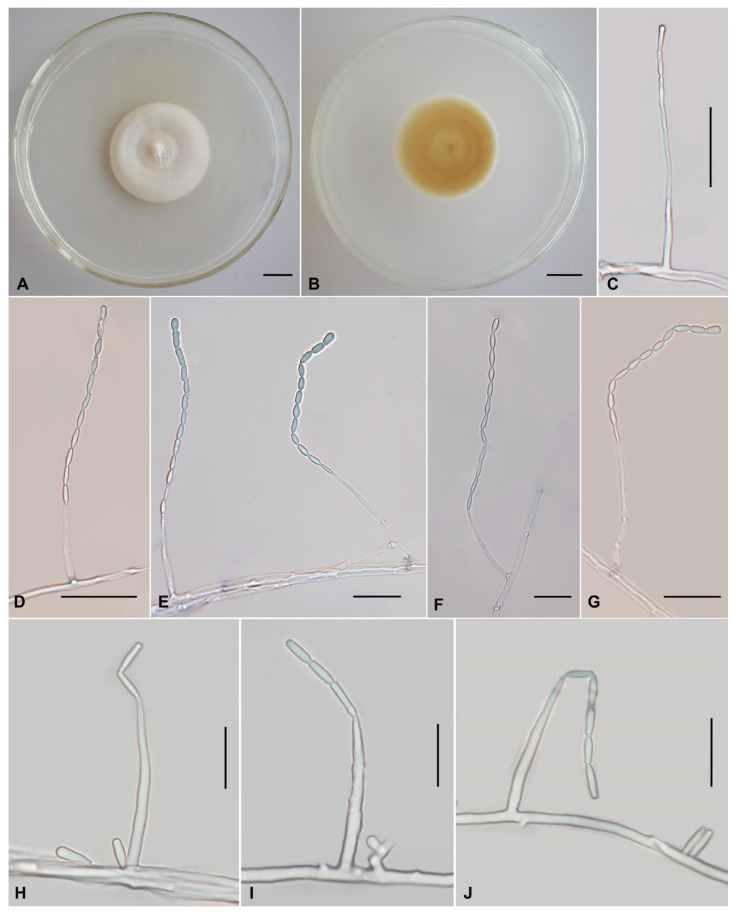
Morphology of *Leptobacillium gasaense*. (**A**,**B**) Colonies on PDA after 21 days ((**A**) obverse; (**B**) reverse). (**C**–**J**) Solitary phialides with conidia in chains are produced on prostrate aerial hyphae. Scale bars: (**A**,**B**) = 10 mm; (**C**–**G**) = 20 µm; (**H**–**J**) = 10 µm.

**Figure 3:**
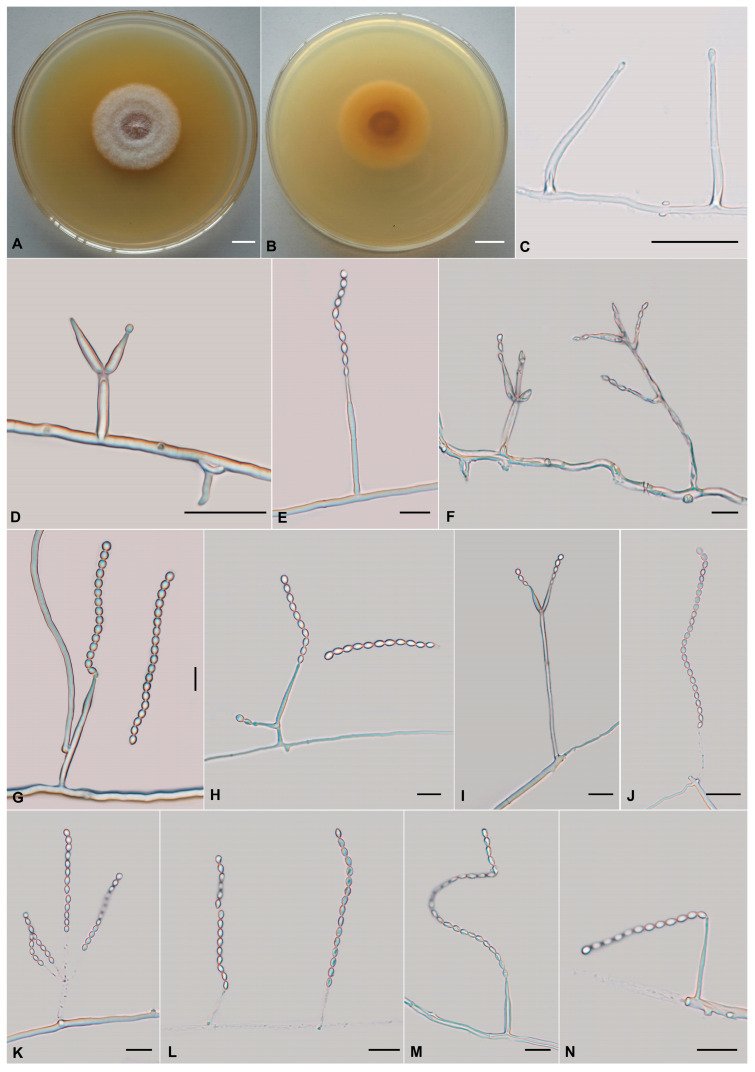
Morphology of *Muscodor coffeanus*. (**A**,**B**) Colonies on PDA after 1 month ((**A**) obverse; (**B**) reverse). (**C**–**N**) Phialides and conidia. Scale bars: (**A**,**B**) = 10 mm; (**C**,**D**,**G**,**H**,**J**) = 20 µm; (**E**,**F**,**I**,**K**–**N**) = 10 µm.

**Figure 4:**
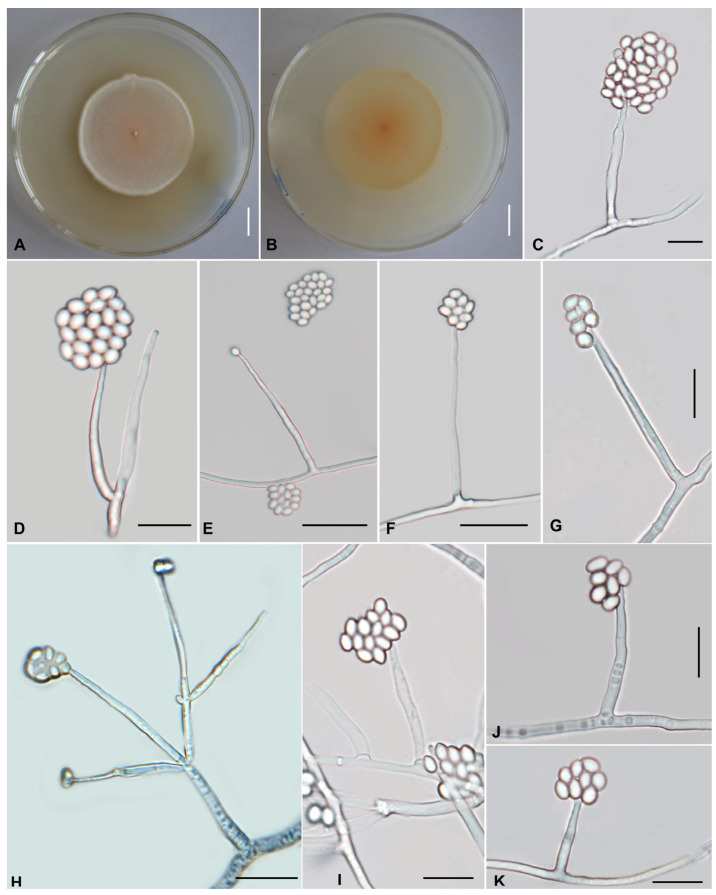
Morphology of *Ovicillium yunnanense*. (**A**,**B**) Colonies on PDA after 21 days ((**A**) obverse; (**B**) reverse). (**C**–**K**) Phialides and conidia. Scale bars: (**A**,**B**) = 10 mm; (**C**,**D**,**G**,**I**–**K**) = 10 µm; (**E**,**F**,**H**) = 20 µm.

The morphological characteristics of those three described species (Cordycipitaceae: *L*. *gasaense*, Xylariales: *M*. *coffeanus,* and Bionectriaceae: *O*. *yunnanens*), as well as morphological structures in photomicrographs, are shown in Figure 2, Figure 3 and Figure 4. The detailed fungal morphological descriptions are provided in the Section 3.3.

### 3.3. Taxonomy

Two new species and a new record are described in this study.

***Leptobacillium*** Zare & W. Gams, Mycol. Progr. 15: 1001 (2016).

*Systematic position*: Fungi, Dikarya, Ascomycota, Pezizomycotina, Sordariomycetes, Hypocreomycetidae, Hypocreales, Cordycipitaceae

*Type species*: ***Leptobacillium leptobactrum*** (W. Gams) Zare & W. Gams, Mycol. Progr. 15: 1003 (2016).

Table 2 lists all hosts, substrates, and geographical locations of *Leptobacillium* species. Table 3 reveals differences with asexual morphs, including the conidial shapes and conidiogenous structures of known species in *Leptobacillium*.

**Table 2:** *Leptobacillium* hosts, substrates, and geographical location.

No.	Species	Host/Substrate	Countries Found	References
1	*L. cavernicola*	Air	France	[20]
2	*L. chinense*	Wood submerged in freshwater, environmental microorganisms	China	[18]
3	*L. coffeanum*	*Coffea arabica* (branches), *Ophiocordyceps nutans*, soil	China, Brazil	[21,64]
4	*L. filiforme*	Endophyte from *Citrullus lanatus*, *Thozetella pindobacuensis*	Brazil	[37]
**5**	***L. gasaense***	***Gibellula*** **sp.**	**China**	**This study**
6	*L. latisporum*	Soil	Thailand	[23]
7	*L. leptobactrum*	Decaying wood, *Lactarius rufus*, *Phlebia tremellosa*, human nail, sandy soil, unknown ascomycete	Poland, France, Iran, Netherlands	[6]
8	*L. leptobactrum* var. *calidius*	Living lepidopterous larva, cyst of *Heterodera glycines*, *Hemileia vastatrix* on Coffea	Ghana, USA, Brazil, France, Netherlands	[6]
9	*L. leptobactrum* var. *leptobactrum*	Lepidoptera larva	Ghana, Poland, France, Iran, Netherlands	[6]
10	*L. muralicola*	On acrylic varnish coatings of murals	China	[22]
11	*L. symbioticum*	From sori of soybean rust fungus	Japan	[19]
12	*L. xianyushanense*	*Camellia oleifera* rhizosphere soil	China	[24]

**Table 3:** Morphological comparisons of asexual morphs in *Leptobacillium*.

No.	Species	Colony	Phialides (µm)	Conidia (µm)	References
1	*L. cavernicola*	White, reverse dark brown	Solitary, 5.1–27.2 × 1.2–1.7	Narrowly cylindrical to slightly fusiform, 3.1–6.9 × 0.9–1.5	[20]
2	*L. chinense*	White, reverse cream-colored to light yellow	Solitary, (6.0–) 15–30 (–68.0) × 1.5	Mostly oval, ellipsoidal or cylindrical, 3.5–5.0 × 1.0–1.5; the apical conidia of the conidial chains subglobose to obovoid, 1.5–2.5 × 1.5–2.0	[18]
3	*L. coffeanum*	White, reverse cream-colored	Solitary, rarely in whorls of 2–3, 11–44 (–70) × 1.0–2.4	Macroconidia spindle-shaped, 5.3–8.8 × 1.0–1.6 µm; Microconidia ellipsoidal to fusiform, 2.2–3.8 × 0.8–1.5	[21]
3	*L. coffeanum*	White to cream, reverse orange-yellow	Solitary or in whorls of 2–3, 13.7–81.1 × 1.8–2.9	Macroconidia spindle-shaped, 3.3–6.2 × 1.2–3.2; Microconidia ellipsoidal to fusiform, 2.9–4.1 × 1.4–2.2	[64]
4	*L. filiforme*	White, reverse white to yellowish cream	Solitary, 9–18 × 1	Fusoid to filiform, catenulate, sometimes forming zigzag chains, 7.2–12.5 × 1	[37]
**5**	***L. gasaense***	**White to cream, reverse pale luteous, yellow to yellowish brown**	**Solitary, rarely in whorls of 2–3, 12.2–30.3 × 1.3–2.5**	**Bacilliform or narrowly cylindrical (rod-shaped), 3.9–6.8 × 0.9–2.5; the apical conidia of the conidial chains, fusiform, subglobose to obovoid, 2.7–3.8 × 1.8–2.6**	**This study**
6	*L. latisporum*	White, reverse grayish orange to orange-white at the margin	Solitary or in whorls of 2–3, cylindrical, 13.2–40.8 × 3–4.8	Slightly fusoid to narrowly cylindrical, 4–6.3 × 1.9–3.8	[23]
7	*L. leptobactrum* var. *calidius*	White to cream, reverse pale yellow to brown	Solitary, rarely in whorls of 1–2, 18.4–60.0 × 0.7–2.0	Narrowly cylindrical (rod-shaped) to slightly fusiform, 3.0–5.7 × 0.7–1.7	[64]
8	*L. leptobactrum*	White, grayish white to pinkish white, reverse orange to orange-brown, ochraceous, pale luteous, milky white to dark buff	Solitary, rarely in whorls of 1–2, 20–45 µm long, 1–2 µm wide (base) to 0.5–0.7 µm wide (apex)	Narrowly cylindrical (rod-shaped) to slightly fusiform, 4.5–8 × 0.8–1.5 (–2)	[6]
9	*L. Leptobactrum* var. *leptobactrum*	White to cream, reverse light yellow to yellowish brown	Solitary, rarely in whorls of 2–3, 15.8–31.7 × 0.7–1.5	Narrowly clavate or narrowly cylindrical (rod-shaped), 3.0–6.1 × 0.8–2.1	[64]
10	*L. muralicola*	White, grayish white to greenish white, reverse pale luteous, milky white to dark buff, orange to orange-brown, ochraceous	Solitary, rarely in whorls of 1–2, 20–45 µm long, 1–2 µm wide (base) to 0.5–0.7 µm wide (apex)	Narrowly cylindrical (rod-shaped) to slightly fusiform, 4.5–6 × 1–2	[22]
11	*L. symbioticum*	White, reverse orange-yellow to orange-brown	Solitary, rarely in whorls of 2–3, 7.1–30.6 × 1.6–3.5	Slightly fusiform to narrowly cylindrical, 4.0–6.9 × 0.7–1.6	[19]
12	*L. xianyushanense*	White, irregular floccose surface with divergent cracks, reverse orange to orange-brown, ochraceous, pale luteous	Solitary, rarely with branches of two, tapering 24.7–28.5 × 1.5–1.9	Narrowly cylindrical (rod-shaped) to slightly fusiform. 3.8–5.6 × 0.5–1.2	[24]

Key to the species of *Leptobacillium*
1a.Phialide simple, up to two whorls.....................…………………………………………………………..21bPhialide simple, 2–3 whorls……….......................…………………………………………………………..62aConidia cylindrical (rod-shaped) to slightly fusiform…………………………………..................………………...…….32bConidia oval, ellipsoidal or filiform………………………………………………......................................……......………53aPhialide > 30 µm long; conidia relatively bigger (4.5–8 × 0.8–1.5(–2) µm)…….....……….……………***L. leptobactrum***3bPhialide < 30 µm long; conidia relatively smaller……………………………….................………………………………44aPhialide relatively narrower, 1.2–1.7 µm………………………………………..……..…………....…...…***L. cavernicola***4bPhialide relatively wider, 1.5–1.9 µm………...……………………………................……………...….***L. xianyushanense***5aPhialide relatively longer, (6.0–) 15–30 (–68.0) × 1.5 µm…………………….……………………………....…***L. chinense***5bPhialide relatively shorter, 9–18 × 1 µm…………………………………..……………………………..………***L. filiforme***6aPhialide > 50 µm long……………………...........………………..…... ……………………………………………..……….76bPhialide < 50 µm long……………………………......…………….………………….…………………………...………….77aConidia spindle-shaped, 3.3–6.2 × 1.2–3.2 µm……...………….…………………………………….......……***L. coffeanum***7bConidia narrowly cylindrical (rod-shaped) to slightly fusiform, 3.0–5.7 × 0.7–1.7 µm……………………………......………………………………………………………….…***L. leptobactrum* var. *calidius***8aIsolated from inorganic substrate (acrylic varnish coatings of murals, soil)…....................................………….........…98bIsolated on insecta (larvae, Lepidoptera) or fungi………………………………………………………….……..........…109aPhialide relatively longer, 20–45 µm long; conidia relatively narrower, 1–2 µm ……..........................…***L. muralicola***9bPhialide relatively shorter, 13.2–40.8 µm long; conidia relatively wider, 1.9–3.8 µm……….…..…........***L. latisporum***10aOn Lepidoptera larva…...….………….……………………...………………………..…..………...…***L.* var*. leptobactrum***10bOn fungi……………………....……………………………......……………………………......…………….………………1111aIsolated from *Gibellula* sp., conidia relatively wider, 0.9–2.5 µm...….........................................................…..***L. gasaense***11bFrom sori of soybean rust fungus, conidia relatively narrower, 0.7–1.6 µm………………...........……..…***L. symbioticum***

***Leptobacillium gasaense*** Q.Y. Dong **sp. nov.**

Figure 2

MycoBank: 857486

*Etymology*: The name reflects the location of Gasa County, where the species was isolated.

*Holotype*: China, Yunnan Province, Jinghong City, Gasa County, Anmalaozhai Village (22°08′53″ N, 100°40′01″ E, alt. 690 m), isolated from the *Gibellula* sp., 29 July 2024, Quanying Dong, dried culture on PDA (holotype CXTC 0003; ex-holotype living culture, CXCC 0003).

*Sexual morph*: Undetermined.

*Asexual morph*: Colonies on PDA are moderately fast-growing, attaining a diameter of 34–37 mm in 21 days at 25 °C. Colonies cotton, fluffy, with high mycelial density, white to cream, reverse pale luteous, yellow to yellowish brown. Hyphae smooth-walled, branched, septate, hyaline, 1.3–2.5 µm wide. Cultures produce phialides and conidia on PDA after 10 days at room temperature. Phialides arising from aerial hyphae, usually solitary, rarely in whorls of two to three, 12.2–30.3 × 1.3–2.5 µm, 1.4–2.5 µm wide at the base, 0.9–1.3 µm wide at the top, long cylindrical, tapering gradually toward the apex. Conidia hyaline, one-celled, narrow clavate or narrowly cylindrical (rod-shaped), single or arranged in very long, slender chains at the apex of phialides, 3.9–6.8 × 0.9–2.5 µm. The first-formed conidium is usually shorter, ellipsoid, or with a rounded distal end, 2.7–3.8 × 1.8–2.6 µm. Chlamydospores not observed.

*Substrate*: *Gibellula* sp. (Cordycipitaceae)

*Known distribution*: Yunnan Province, China.

*Additional specimens examined*: China, Yunnan Province, Jinghong City, Gasa Country, Anmalaozhai Village (22°08′53″ N, 100°40′01″ E, alt. 690 m), isolated from the *Gibellula* sp., 29 July 2024, Quanying Dong, dried culture on PDA (paratype CXTC 0004; ex-paratype living culture CXCC 0004).

*Commentary*: Our phylogenetic analysis indicates that *Leptobacillium gasaense* is closely related to *L. latisporum* TBRC 16288 (BS = 70% and PP = 0.85), which was firstly isolated from soil in Thailand, mainly produces long solitary phialides, rarely in whorls of 2–3, 13.2–40.8 × 3–4.8 µm, conidia lisghtly fusoid to narrowly cylindrical, 4–6.3 × 1.9–3.8 µm [23]. Morphologically, *L. gasaense* differs from *L. latisporum* in the following aspects. Relatively*, L. gasaense* has thinner phialides (12.2–30.3 × 1.3–2.5 µm vs. 13.2–40.8 × 3–4.8 µm) and narrower conidia (3.9–6.8 × 0.9–2.5 µm vs. 4–6.3 × 1.9–3.8 µm). The molecular divergence between *L. gasaense* and *L. latisporum*, based on ITS and LSU sequence data. *Leptobacillium gasaense* vs. *O. attenuatum*: ITS: 85 bp differences. LSU: 17 bp differences.

***Muscodor*** Worapong, Strobel & W.M. Hess, Mycotaxon 79: 71 (2001)

*Systematic position*: Fungi, Dikarya, Ascomycota, Pezizomycotina, Sordariomycetes, Xylariomycetidae, Xylariales

*Type species: **Muscodor albus*** Worapong, Strobel & W.M. Hess 2001

***Muscodor coffeanus*** A.A.M. Gomes, Pinho & O.L. Pereira [as ‘*coffeanum*’], Cryptog. Mycol. 36(3): 368 (2015)

Figure 3

*Sexual morph*: For detailed descriptions and images of *M. coffeanus,* see Li and Kang [65].

*Asexual morph*: Colonies on PDA are slowly growing, attaining a diameter of 37–40 mm after 21 days at 25 °C. Colonies white to pale yellow, with high mycelial density, flocculose. Reverse yellow to brown. Hyphae hyaline, branched, smooth-walled, septate, 1.2–2.4 µm wide. Cultures readily produced phialides and conidia on PDA after four months at 25 °C. Conidiophores cylindrical, hyaline, smooth-walled, solitary or verticillate, 15.4–55.8 × 1.1–2.6 µm, 1.4–2.4 µm wide (apex), 1.1–2.2 µm wide (base). Phialides from aerial mycelium, straight to slightly flexuose, solitary or in whorls of two to five on each branch, cylindrical, usually with a slightly swollen basal part, tapering into the apex form a long neck, 6.9–34.7 × 1.6–2.5 µm, 0.3–1.5 µm wide (apex), 1.4–2.5 µm wide (base). Conidia one-celled, hyaline, smooth, ovoid to ellipsoidal, globose to subglobose; the conidia exhibit catenulate arrangement, producing distinct chains, 2.0–3.2 × 1.5–2.5 µm. Chlamydospores not observed.

*Known distribution*: Brazil, China, Thailand [42,65].

*Specimens examined*: China, Sichuan Province, Dujiangyan City, Qingcheng Hou Mountain, Youyi Village (30°56′10″ N, 103°28′19″ E, alt. 1270 m), isolated from the *Ophiocordyceps* sp., 19 August 2023, Quanying Dong, dried culture on PDA (CXTC 0001, living culture CXCC 0001; CXTC 0002, living culture CXCC 0002).

*Commentary*: *Muscodor coffeanus* as an endophytic fungus with a full description and illustration of the sterile mycelium described from Brazil by Hongsanan et al. [42], and subsequently collected from Thailand, is isolated from a deadwood piece of an unidentified plant with sexual morphological characteristics and phylogenetic analyses results [65]. VOCs, including cyclosativene compound and phytase, have been detected in the culture of *M*. *coffeanus* [66]. *Muscodor coffeanus* produces VOCs with antifungal activity against *Botrytis cinerea* Pers. and antibacterial activity against *Staphylococcus aureus* Rosenbach, *Enterococcus faecalis* (Andrewes and Horder) Schleifer and Kilpper-Bälz, and *E. faecium* (Orla-Jensen) Schleifer and Kilpper-Bälz; moreover, anti-nematode activity against *Meloidogyne incognita* (Kofoid & White) Chitwood [15,66,67].

In our two-locus (ITS and nrLSU) phylogenetic analysis, *M. coffeanus* is, with strong support (BS = 86% and PP = 0.82), related to *M. yucatanensis* (M.C. González, A.L. Anaya, Glenn & Hanlin) Samarak. et al.; the four strains (COAD 1842, COAD 1900, CXCC 0001 and CXCC 0002) formed a distinct lineage. COAD 1842 as the type material of *M. coffeanus* from Brazil. Since no significant ITS sequence differences were found between the Chinese collections and that of Brazil, we treated CXCC 0001 and CXCC 0002 as *Muscodor coffeanus*, a new record for China based on its mycelial characteristics, asexual morphology, and phylogenetic analyses. *Muscodor coffeanus* is easily distinguished by its rope-like hyphal bodies and coil-like structure; solitary or verticillate, cylindrical conidiophores; solitary or in whorls of two to five, cylindrical phialides and ovoid to ellipsoidal, globose to subglobose conidia. Moreover, this report is the first study on asexual characters of *Muscodor* genus.

***Ovicillium*** Zare & W. Gams, Mycol. Progr. 15: 1020 (2016)

*Systematic position*: Fungi, Dikarya, Ascomycota, Pezizomycotina, Sordariomycetes, Hypocreomycetidae, Hypocreales, Bionectriaceae

*Type species*: ***Ovicillium attenuatum*** Zare & W. Gams, Mycol. Progr. 15: 1021 (2016)

Table 4 lists the hosts, substrates, and geographical locations of all *Ovicillium* species. Table 5 shows the differences between the asexual morphs, including conidiogenous structures, conidial shapes, and chlamydospores of known species in *Ovicillium.*

**Table 4:** Hosts, substrates, and geographic distribution of *Ovicillium*.

No.	Species	Host/Substrate	Countries Found	References
1	*O. asperulatum*	Forest soil	Spain	[26]
2	*O. attenuatum*	*Auricularia* sp.	Cuba, Ecuador, Papua New Guinea	[6]
3	*O. oosporum*	*Theobroma gileri, Grandinia pallidula, Theobroma* sp., human, *Hypholoma* sp, *Leptomitus lacteus*, *Fomitopsis pinicola,* soil under *Elaeis guineënsis, Xylaria* sp. on log	South America (Brazil?), Belgium, Ecuador, France, Netherlands, Poland, Surinam, USA	[6]
4	*O. sinense*	Lepidoptera pupa	China	[27]
5	*O. subglobosum*	Soil, *Theobroma gileri*	China, Brazil, Puerto Rico	[6]
6	*O. variecolor*	Forest soil	Spain	[26]
**7**	***O. yunnanense***	**Dead insect on leaf**	**China**	**This study**

**Table 5:** Morphological comparisons of asexual morphs in *Ovicillium*.

No.	Species	Colony	Conidiophores	Phialides (µm)	Conidia (µm)	Chlamydospores	References
1	*O. asperulatum*	Dark, white or yellowish-white, reverse yellowish or amber yellow	Solitary or in whorls of 2–4, up to 105 µm long	Acicular, 28–68 µm long, 1–2 µm wide at the base	Globose, 3–4 (–5) µm diam, chromophilic, arranged in slimy heads	Present	[26]
2	*O. attenuatum*	White, dirty white to pinkish, ochraceous to pale ochraceous, light hazel to buff, reverse pinkish to pale white	Solitary and verticillate	Aculeate, 25–50 × 1.7–3.3	Oval to subglobose, strongly cyanophilic, 3.5–5 × 2.5–3.8, aggregated in large globose to subglobose heads	Absent	[6]
3	*O. oosporum*	Grayish to dark buff, light honey to hazel, reverse white, pale brown, gray to grayish white, pale yellow to brown	Solitary or in whorls of 2–5, 20–50 × 1.2–2.2	–	Subglobose, oval to broadly oval, cyanophilic, 4–6 × 2.5–4, aggregated in large globose heads	Present or absent	[6]
4	*O. sinense*	White, reverse yellowish	Solitary or in whorls of 2–5, 17.0–21.7 × 2.3–3.0	Cylindrical, 16.2–25.8 × 1.7–2.4	Globose to ovoid, 2.1–2.9 × 1.1–1.7, aggregated in large globose to subglobose heads	–	[27]
5	*O. subglobosum*	Grayish buff to dark buff, light smoke-gray to light hazel, reverse pale gray, dirty white to grayish cream-colored	Solitary or in whorls of 2–4	25–55 × 1.5–2.2	Subglobose (or nearly globose), rather cyanophilic, 3.5–5.5 × 3.5–4.5	Absent	[6]
6	*O. variecolor*	Yellowish white to grayish yellow	Solitary or in whorls of 2–5, 290 µm long	Acicular, 18–95 µm long, 1–2 µm wide at the base	Subglobose or ovoid, 3–4 (–5) × 2–4, arranged in slimy heads; sessile conidia solitary, cylindrical or ellipsoidal, 5–7 (–9) × 2–3 (–4)	Absent	[26]
**7**	***O. yunnanense***	**White to pale yellowish-orange, reverse yellow to brown**	**Solitary or in whorls of 2–5**	**Cylindrical, 22.7–87.8 × 1.4–3.3**	**Subglobose, ovoid to ellipsoidal, 2.1–4.4 × 1.9–3.8, aggregated in large globose to subglobose heads**	**Absent**	**This study**

Key to the species of *Ovicillium*
1a.Conidia globose...............................................................................................................................................***O. asperulatum***1b.Conidia oval to subglobose......................................................................................................................................................22a.Isolated from dead insect on leaf....................................................................................................................***O. yunnanense***2b.Isolated from other substrate or host......................................................................................................................................33a.Conidia > 3.0 µm long…………………………….................................................................…………….....................…….43b.Conidia < 3.0 µm long ( 2.1–2.9 × 1.1–1.7 µm)……................................................................………..………….***O. sinense***4a.Chlamydospores scarce if present, widespread geographical distribution………………..…..………....***O. oosporum***4b.Chlamydospores absent, limited geographical distribution…….........................................................……........…….55a.From soil, phialides relatively longer ……….....………...……..........................................................................……...…...65b.From *Auricularia* sp, phialides relatively shorter (25–50 × 1.7–3.3 µm).....................................................***O. attenuatum***6aConidiophores solitary or in whorls of 2–4 phialides, phialides relatively shorter (25–55 µm)....................................................................................................................................................................***O. subglobosum***6bConidiophores solitary or in whorls of 2–5 phialides, phialides relatively longer (18–95 µm).........................................................................................................................................................................***O. variecolor***

***Ovicillium yunnanense*** Q.Y. Dong, **sp. nov.**

Figure 4

MycoBank: 857488

*Etymology*: The name reflects the location of Yunan Province, where the species was isolated.

*Holotype*: China, Yunnan Province, Jinghong City, Gasa County, Huilaoxiaozhai Village (22°09′14″ N, 100°41′13″ E, alt. 670 m), isolated from dead insect on leaf, 29 July 2024, Quanying Dong, dried culture on PDA (holotype CXTC 0005; ex-holotype living culture CXCC 0005).

*Sexual morph*: Undetermined.

*Asexual morph*: Colonies on PDA are fast-growing, attaining a diameter of 46–48 mm after 21 days at 25 °C. Colonies white to pale yellowish orange, margin thick, with high mycelial density, pulvinate, asperulate. Reverse yellow to brown. Hyphae hyaline, branched, smooth-walled, 1.4–3.1 µm wide. Cultures readily produced phialides and conidia on potato dextrose agar after 7 days at 25 °C. Conidiophores are cylindrical, hyaline, smooth-walled, simple to verticillate form 1–3. Phialides from aerial mycelium straight to slightly flexuose, solitary or in whorls of two to five on each branch, cylindrical, usually with a slightly swollen basal part, tapering into the apex from a long neck, 22.7–87.8 × 1.4–3.3 µm, 1.4–2.2 µm wide (apex), 1.8–2.3 µm wide (base). Conidia one-celled, hyaline, smooth, subglobose, ovoid to ellipsoidal, 2.1–4.4 × 1.9–3.8 µm, aggregated in large globose to subglobose heads. Chlamydospores not observed.

*Other material examined*: China, Yunnan Province, Jinghong City, Gasa County, Huilaoxiaozhai Village (22°09′14″ N, 100°41′13″ E, alt. 670 m), isolated from a dead insect on a leaf, 29 July 2024, Quanying Dong, dried culture on PDA (paratype CXTC 0006; ex-paratype living culture CXCC 0006).

*Habitat*: Dead insect on leaf.

*Known Distribution*: Yunnan Province, China.

*Commentary*: *Ovicillium yunnanense* displays characteristic genus-level features consistent with other *Ovicillium* species, including solitary or whorled phialides (2–5 per node) and conidia varying from subglobose to ovoid or ellipsoid in shape. The species can be distinguished by the following unique combination of morphological characteristics: solitary or in whorls of 2–5, cylindrical phialides, 22.7–87.8 × 1.4–3.3 µm; and mostly subglobose, ovoid to ellipsoidal conidia, 2.1–4.4 × 1.9–3.8 µm, conidia aggregated in large globose to subglobose heads. Furthermore, it is isolated from a dead insect on a leaf. It is phylogenetically, with high support (BS = 80%, PP = 0.79), clustered with *O. attenuatum* and *O. sinense*, but it is distinguished from the two latter species by forming a separate clade in this group (Figure 1). Morphologically, these two species differ from *O. yunnanense* in the following ways. *Ovicillium attenuatum*, a species described from Cuba, Ecuador, and Papua, New Guinea, has an aculeate and relatively shorter phialides measuring 25–50 × 1.7–3.3 µm [6]. *Ovicillium sinense,* a species described from Guizhou Province, Duyun City, and also similar to *O. yunnanense* in appearance, has a relatively shorter phialide (16.2–25.8 × 1.7–2.4 µm vs. 22.7–87.8 × 1.4–3.3 µm), and relatively smaller conidia (2.1–2.9 × 1.1–1.7 µm vs. 2.1–4.4 × 1.9–3.8 µm) [27]. The molecular divergence between *O. yunnanense*, *O. attenuatum,* and *O. sinense*, based on ITS and LSU sequence data. *O. yunnanense* vs. *O. attenuatum*: ITS: 7 bp differences. LSU: 0 bp differences. *O. yunnanense* vs. Ovicillium sinense: ITS: 13 bp differences. LSU: 2 bp differences.

## 4. Discussion

### 4.1. Species Diversity of Leptobacillium and Ovicillium

In the past five years, significant taxonomic advancements have been made within the order Hypocreales, with numerous new species described [8,9,20,64,68,69,70]. This study contributes to this progress by reporting the discovery of a new Leptobacillium species and a new Ovicillium species. To date, 12 taxa (comprising 10 species and 2 varieties) have been documented globally within the genus Leptobacillium (Index Fungorum, accessed 20 February 2025; https://www.indexfungorum.org/Names/Names.asp).

Leptobacillium species exhibit a wide range of substrate preferences, from broad to narrow. For example, L. leptobactrum and its variety L. leptobactrum var. calidius are notable for their broad substrate adaptability. Members of this genus are primarily isolated from soil and are morphologically characterized by the production of solitary or whorled phialides (typically in groups of 2–3) and narrowly cylindrical (rod-shaped) to slightly fusiform conidia (Table 2).

In contrast, all known species of *Ovicillium* exist exclusively in their asexual states in nature, with no sexual states observed to date. The genus exhibits distinct biogeographic patterns: while some species demonstrate cosmopolitan distributions, others show restricted endemic ranges, particularly in China and Spain (Table 4). Our phylogenetic reconstruction reveals a fundamental evolutionary split within *Ovicillium*, delineating two well-supported clades: a basal lineage comprising European taxa (*O. asperulatum, O. oosporum, O. subglobosum* and *O. variecolor*), predominantly isolated from soil substrates and characterized by dark-pigmented mycelia; a derived clade containing three Asian species (*O. sinense* and *O. yunnanense* and tropical-distribution *O. attenuatum*) isolated from more specialized substrates, including fungal and insect hosts, characterized by grayish-pigmented mycelia. Across the genus, *Ovicillium* species demonstrate broad ecological versatility, having been recovered from diverse substrates such as soil, decaying plant material, and various environmental samples. Morphologically, Ovicillium species are distinguished by the production of solitary or whorled phialides (typically in groups of 2–5), which are acicular to cylindrical in shape. Their conidia are oval to subglobose and often aggregate into large globose to subglobose heads (Table 5).

### 4.2. Species Delimitation in Muscodor

In fungal taxonomy, the morphological characteristics of asexual microfungi are often limited, leading to significant challenges in species’ identification. The presence of distinctive and diagnostically informative macro- and microscopic features is essential for accurate classification of species. Historically, morphological traits have played a central role in taxonomic research, with most fungal species described to date relying on morphological criteria. These traits not only aid in discrimination between species but also provide insights into evolutionary relationships, particularly within higher taxonomic groups such as Mucoromycota, Ascomycota, and Basidiomycota, where differences in sexual sporulation structures are often diagnostic. Additionally, the evolutionary history of major fungal taxa can be inferred through trends in morphological traits and fossil evidence. In Ascomycota, taxonomic classification relies on distinct morphological features, including asexual features—such as conidiophore structure, phialide morphology, and conidial shape, size, and pigmentation—or sexual characteristics, including perithecia, asci, ascospores, part-spores, and their morphological variations in size, ornamentation, and arrangement. Collectively, these features form the foundation for delineating species and understanding their phylogenetic relationships.

To date, 27 species of Muscodor have been formally described (Index Fungorum, accessed 20 February 2025; https://www.indexfungorum.org/Names/Names.asp), with delineation of species based on a combination of culture characteristics, VOC profiles, and molecular phylogenetic analyses. Notably, prior to this study, no conidiogenous structures had been observed in Muscodor, and taxonomic characterization primarily relied on colony morphology and mycelial features. Although some species exhibit distinctive hyphal arrangements [39,71], these traits are not diagnostically reliable. The most significant taxonomic feature in Muscodor is likely its conidiogenous structures, which have remained elusive until now.

The unique profiles of volatile metabolites produced by Muscodor species, analyzed through gas chromatography–mass spectrometry (GC–MS), have been widely used as a criterion for classification [72]. Several studies have employed VOC production as a key feature for species’ identification [15,39,47]. Samarakoon et al. [12] highlighted the potential of secondary metabolite profiles for chemotaxonomic purposes but emphasized the need for rigorous standardization, as metabolite production is influenced by culture conditions, growth phase, and the inclusion of a representative number of species.

While no other taxa within Xylariales have been systematically compared based on VOC profiles as extensively as Muscodor species [73], it is important to note that phylogenetic relationships may not always correlate with VOC diversity. Phylogenetic studies in Muscodor have primarily relied on the uniqueness of DNA sequences, including the ITS and nrLSU regions, and protein-coding genes such as rpb2 and tub2. However, for most Muscodor species, ITS rRNA gene sequences remain the only available molecular data, limiting the resolution of phylogenetic relationships within Xylariales [62,73]. Consequently, species’ identification in Muscodor remains ambiguous, and its taxonomic position within Xylariales continues to be debated.

Stadler et al. [73] raised significant doubts regarding the taxonomic definition of Muscodor but did not recommend integrating this younger asexual morph genus into any of the older established genera. Wendt et al. [74] further challenged the validity of Muscodor, suggesting that its classification does not adhere to rigorous taxonomic standards as outlined by Stadler et al. [70]. More recently, Voglmayr et al. [75] rejected the recognition of Induratiaceae as a distinct family, proposing instead its inclusion within Xylariaceae based on phylogenetic and morphological evidence. These ongoing debates underscore the need for more precise and standardized taxonomic practices in fungal systematics [70,76]. Consequently, it is imperative to accelerate the discovery and description of Muscodor species through integrated morphological and molecular approaches. Such efforts will not only clarify the taxonomic status of Muscodor but also contribute to a more robust understanding of its phylogenetic relationships within Xylariaceae.

## Data Availability

The original contributions presented in this study are included in the article. Further inquiries can be directed to the corresponding authors.
